# Supplementary material for: Reconciling Mining with the Conservation of Cave Biodiversity: A Quantitative Baseline to Help Establish Conservation Priorities
Source: PLoS One. 2016 Dec 20;11(12):e0168348. doi: 10.1371/journal.pone.0168348 (PMC5173368; doi:10.1371/journal.pone.0168348)
Supplement: S1 Dataset — (ZIP) [file pone.0168348.s002.zip › Taxa/Serra Norte/SN_2013/N4E/Lista N4E-89.pdf]

CAVIDADE N4E-0089

Classe	Ordem	Fam/Outros	Gên/Outros	Espécie	S-U	U-S	Zon.
Annelida	Oligochaeta			jovem		3	E/P
Arachnida	Acari	(Oribatida)		sp.3		X	P
Arachnida	Acari	(Parasitengonina)		sp.7		X	P
Arachnida	Acari	Neothyridae	*Diplothyrus*	sp.1		X	P
Arachnida	Acari	Prostigmata		sp.49	X		E/P
Arachnida	Acari	Rhagidiidae		sp.5		X	P
Arachnida	Acari	Trombiculidae		sp.39		X	P
Arachnida	Acari	Veigaiidae		sp.1		X	P
Arachnida	Amblypygi	Charinidae	*Charinus*	sp.1	3	5	E/P
Arachnida	Amblypygi	Phrynidae	*Heterophrynus*	*longicornis*	1		A
Arachnida	Araneae	Corinnidae		jovem	1	4	P/A
Arachnida	Araneae	Drymusidae		jovem		X	E/P
Arachnida	Araneae	Ochyroceratidae	*Ochyrocera*	sp.1	X		P
Arachnida	Araneae	Ochyroceratidae	*Speocera*	sp.1	X	X	P/A
Arachnida	Araneae	Pholcidae	*Mesabolivar*	*aurantiacus*	X	X	
Arachnida	Araneae	Pisauridae		jovem		1	E
Arachnida	Araneae	Prodidomidae		jovem		X	P
Arachnida	Araneae	Theridiidae	*Theridion*	sp.2	X		P
Arachnida	Araneae	Theridiidae	*Theridion*	sp.4	X		P
Arachnida	Araneae	Theridiidae		jovem	X	X	E/P
Arachnida	Araneae	Theridiosomatidae	*Plato*	sp.1	X	X	E/P
Arachnida	Opiliones	Escadabiidae		sp.2	X	X	P
Arachnida	Opiliones	Sclerosomatidae	*Prionostema*	sp.1		1	E
Arachnida	Opiliones	Stygnidae	*Protimesius*	*gracilis*	5		E
Arachnida	Pseudoscorpiones	Bochicidae		sp.1	X		E
Arachnida	Pseudoscorpiones	Chthoniidae	*Pseudochthonius*	sp.1	X	X	P
Arachnida	Ricinulei	Ricinoididae	*Cryptocellus*	jovem		1	P
Arachnida	Schizomida	Hubbardiidae	aff. *Rowlandius*	sp.1		X	P
Chilopoda	Geophilomorpha	Schendylidae		sp.2		1	P
Chilopoda	Scolopendromorpha	Cryptopidae	*Cryptops*	jovem		2	E
Chilopoda	Scolopendromorpha	Scolopocryptopidae	*Newportia*	sp.1		2	P
Chilopoda	Scutigeromorpha	Pselliodidae	*Sphendononema*	*guildingii*	1	3	E
Diplopoda	Polydesmida	Chelodesmidae		jovem	2		P
Diplopoda	Polydesmida	Chelodesmidae		sp.3	1	5	E/P/A
Diplopoda	Polydesmida	Cyrtodesmidae		sp.1	X		P
Diplopoda	Polydesmida	Fuhrmannodesmidae		sp.1	X	X	E/P/A
Diplopoda	Polydesmida	Paradoxosomatidae		sp.1		2	E/P
Diplopoda	Polydesmida	Pyrgodesmidae		sp.1	X	X	P
Entognatha	Collembola	Cyphoderidae		sp.1	X		P
Entognatha	Collembola	Entomobryidae		sp.1	X		A
Entognatha	Collembola	Isotomidae		sp.1	X	X	E/P
Entognatha	Collembola	Isotomidae		sp.2	X		E
Entognatha	Collembola	Paronellidae		sp.1	X	X	E
Entognatha	Collembola	Paronellidae		sp.4	X	X	E/P/A
Entognatha	Collembola	Sminthuroidea		sp.2	X	X	P
Entognatha	Diplura	Campodeidae		sp.1		X	P
Entognatha	Diplura	Japygidae		sp.1		X	P
Insecta	Blattodea			jovem	1	5	E/P/A
Insecta	Coleoptera	Carabidae		sp.3	X	X	E/P

Insecta	Coleoptera	Chrysomelidae		sp.18		X	E
Insecta	Coleoptera	Curculionidae		sp.1		X	P
Insecta	Coleoptera	Dytiscidae		sp.7		X	P
Insecta	Coleoptera	Staphylinidae	Pselaphinae	sp.2		X	E
Insecta	Coleoptera	Staphylinidae	Pselaphinae	sp.20		X	E
Insecta	Diptera	Chironomidae		sp.	X		E
Insecta	Diptera	Drosophilidae		sp.	X		
Insecta	Diptera	Psychodidae	Phlebotominae	sp.	X	X	E/P/A
Insecta	Diptera	Tipulidae	Tipulinae	sp.	X	X	E
Insecta	Heteroptera	Cydnidae		sp.		X	P
Insecta	Heteroptera	Tingidae		jovem	X		E
Insecta	Homoptera	Cixiidae		sp.1	X	X	E
Insecta	Homoptera	Cixiidae		sp.4		X	P
Insecta	Hymenoptera	Formicidae	*Camponotus*	sp.1	X		E
Insecta	Hymenoptera	Formicidae	*Camponotus*	sp.2		X	P
Insecta	Hymenoptera	Formicidae	*Pachycondyla*	sp.1	X	X	E/P
Insecta	Hymenoptera	Formicidae	*Pachycondyla*	sp.2		X	P
Insecta	Hymenoptera	Formicidae	*Paratrechina*	sp.1		X	E
Insecta	Hymenoptera	Formicidae	*Solenopsis*	sp.1	X		E
Insecta	Isoptera	Termitidae	*Nasutitermes*	sp.	X		E
Insecta	Lepidoptera	Noctuoidea		sp.5	1		E
Insecta	Lepidoptera			jovem	X	X	E/P
Insecta	Mantodea	Mantidae		sp.1	1		
Insecta	Orthoptera	Phalangopsidae	*Paraclodes*	sp.	1		E
Insecta	Orthoptera	Phalangopsidae	*Phalangopsis*	sp.	70	60	P/A
Insecta	Psocoptera	Epipsocidae	*Epipsocus*	sp.2	X		E/P
Insecta	Thysanura	Nicoletiidae		sp.1		X	P
Gastropoda	Pulmonata	Bulimulidae	*Naesiotus*	sp.	X	X	P
Gastropoda	Pulmonata	Subulinidae		sp.4		X	A
Gastropoda	Pulmonata	Systrophiidae	*Happia*	sp.		X	E/A
Malacostraca	Isopoda	Armadillidae		sp.1	X	X	E/P
Malacostraca	Isopoda	Calabozoidea		sp.1		X	E
Malacostraca	Isopoda	Dubioniscidae		sp.1		X	P
Malacostraca	Isopoda	Philosciidae		sp.1	X	X	P
Malacostraca	Isopoda	Platyarthridae		sp.1	X	X	E/P
Malacostraca	Isopoda	Scleropactidae	*Circoniscus*	*buckupi*		X	P
Symphyla		Scutigerellidae		sp.		X	E/P/A
Amphibia	Anura	Strabomantidae	*Pristimantis*	cf. *fenestratus*	2	1	E/P
Mammalia	Chiroptera	Furipteridae	*Furipterus*	*horrens*	2		A
Reptilia	Squamata	Boidae		sp.	1		E
